# Intracellular glucose starvation affects gingival homeostasis and autophagy

**DOI:** 10.1038/s41598-022-05398-2

**Published:** 2022-01-24

**Authors:** Runbo Li, Hirohito Kato, Yoichiro Taguchi, Makoto Umeda

**Affiliations:** Department of Periodontology, Osaka Dental University, 8-1, Kuzuhahanazono-cho, Hirakata, Osaka Japan

**Keywords:** Nutrient signalling, Autophagy, Cell migration, Periodontitis

## Abstract

Human gingival fibroblasts (HGnFs) maintain periodontal tissue homeostasis through active proliferation and migration. Clinically, it is considered that the wound-healing ability of the gingival tissue is maintained even in environments with insufficient supply of nutrients, such as glucose, immediately after periodontal surgery. However, the effects of such glucose-deficient environments on HGnFs remain unclear. This study aimed to investigate the effects of low-glucose environment on HGnFs homeostasis. We evaluated gingival wound healing by examining cell proliferation and migration and collagen synthesis in HGnFs cultured in 100, 50, 25, and 0 mg/dL glucose in vitro. The cellular stress levels were determined by measuring the lactate dehydrogenase (LDH) and reactive oxygen species (ROS) levels. The glucose metabolism of HGnFs in the low-glucose concentrations was studied by measuring glucose transporter type 1 (GLUT1) mRNA expression, glucose uptake assays, lactate and ATP productions. Molecular effects were examined with a focus on the LKB1-AMPK signaling pathway. Autophagy activity in glucose-deprived HGnFs was evaluated by measuring the levels of autophagy-related proteins. Low glucose levels increased cellular stress levels, autophagy activity, and enhanced glucose metabolism through the LKB1-AMPK signaling pathway, providing more ATPs to promote wound healing. Our results regarding glucose transfer suggest the rapid healing of gingival wounds.

## Introduction

Wound healing in gingiva and oral mucosa is faster and more efficient than that in the skin tissue and other mucosal tissues because of potential cellular activity^1^. However, the mechanisms underlying these differences are not completely clear. Human gingival fibroblasts (HGnFs) play a crucial role in this wound healing process^2^. During the wound healing process, HGnFs produce a variety of growth factors during wound healing and are actively involved in granulation tissue formation. HGnFs contribute the synthesis of collagen, which is a complex extracellular matrix that is important for the regeneration of a functional periodontium^3^. All these processes directly consist of the proliferation and migration capacity of HGnFs. As the number of HGnFs are greater, the process of soft tissue wound healing is faster in all these processes. In other words, the capacity of HGnFs to proliferate and migrate determines the activity of wound healing^4^. Therefore, the proliferation and migration of HGnFs are considered to be the main components of wound healing.

HGnFs use the glycolytic pathway to sustain cell proliferation and migration^5^. In the past 20 years, several studies have provided lots of information on the principal role of glucose in cell proliferation, which in turn is highly dependent on glucose metabolism. This mechanism not only provides a source of energy but also supplies metabolites for the biosynthesis of nucleic acids and membrane lipids^6^. Nevertheless, in some cases where the existing vasculature is destroyed, the cells experience stressful microenvironments characterized by low glucose concentrations. For example, the blood supply in periodontal tissues after trauma or periodontal surgery is significantly lower than that in healthy tissues. Just like the high glucose model of the diabetic state, there are many studies that were conducted under different glucose conditions. We have previously conducted some studies under a high glucose model of the diabetic state^7^. However, there are few experimental studies on wound healing under hypoglycemic conditions that occur due to inadequate nutrient supply.

Autophagy can be triggered by a varies of physiological phenomena, such as low nutrition. The role of autophagy is to secure a source of nutrients by self-digestion in order to survive starvation^8^. This autophagy can be induced not only by bacteria infection but also by inflammation in the periodontal tissue^9,10^. However, the relationship between autophagy and the gingival wound healing process has not been investigated.

A trigger for autophagy, the liver kinase B1 (LKB1)-adenosine monophosphate-activated kinase (AMPK) signaling pathway is also known as an essential energy sensor that plays a key role in sensing energy availability in various cells. It produces a series of metabolic adaptation pathways to ensure energy supply and cell survival by reducing or producing ATP. Glucose starvation (absence of glucose) can activate the LKB1-AMPK signaling pathway and increase the presence of reactive oxygen species (ROS)^11,12^. ROS can activate the LKB1-AMPK signaling pathway and induce autophagy and cell death, while the LKB-AMPK signaling pathway can prevent cell death induced by excessive ROS.

Currently, the role of the LKB1-AMPK signaling pathway and autophagy in gingival wound healing, especially under nutritionally deprived conditions, is poorly understood. The purpose of this study was to investigate the effect of poor nutrient conditions on HGnFs homeostasis. It is hoped that this study will contribute to a deeper understanding of the mechanism of gingival wound healing in low-nutrition situations.

## Results

### Cell proliferation was generally inhibited after once promoted

**Figure 1:**
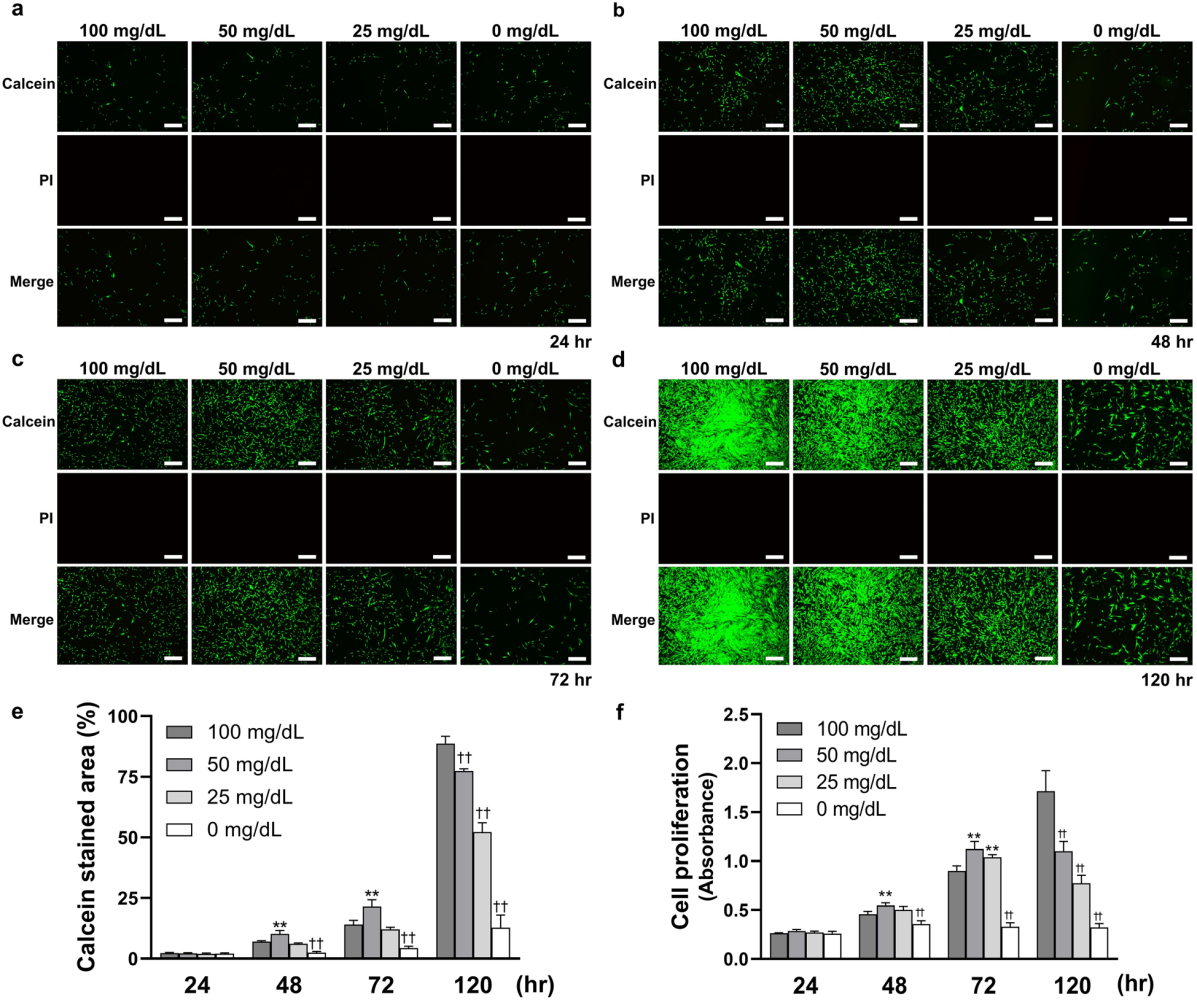
Low glucose concentrations affect the cell proliferation on human gingival fibroblast (HGnFs). (**a**, **b**, **c** and **d**) HGnFs stained by calcein-AM and PI were photographed under a fluorescence microscope after incubation for 24, 48, 72 and 120 h. (**e**) The data of viable and dead staining are presented as the percentage of calcein stained area. (**f**) Cell proliferation was measured after incubation for 24, 48, 72 and 120 h. (Scale bars: 500 μm; A significant increase compared with the control was described as **P < 0.01; A significant decrease compared with the control was described as ^††^*P* < 0.01).

As shown in Fig. 1, the effects of various low glucose concentrations on HGnFs proliferation were measured after culturing for 24, 48, 72, and 120 h. The results showed that at 48 and 72 h, the number of live cells stained with calcein-AM solution was also maximum in 50 mg/dL; At 120 h, the number of live cells decreased proportionally. However, no detectable dead cells were found after staining with PI solution, regardless of the concentration. And HGnFs proliferation was significantly increased in 50 and 25 mg/dL for the first 72 h, with the highest growth observed in 50 mg/dL. The cell proliferation of the 0 mg/dL group was significantly lower than that of the control group (100 mg/dL). At 120 h, HGnFs proliferation in all concentrations, excluding that of the control group, decreased.

### Wound healing capability was generally inhibited after once promoted

**Figure 2:**
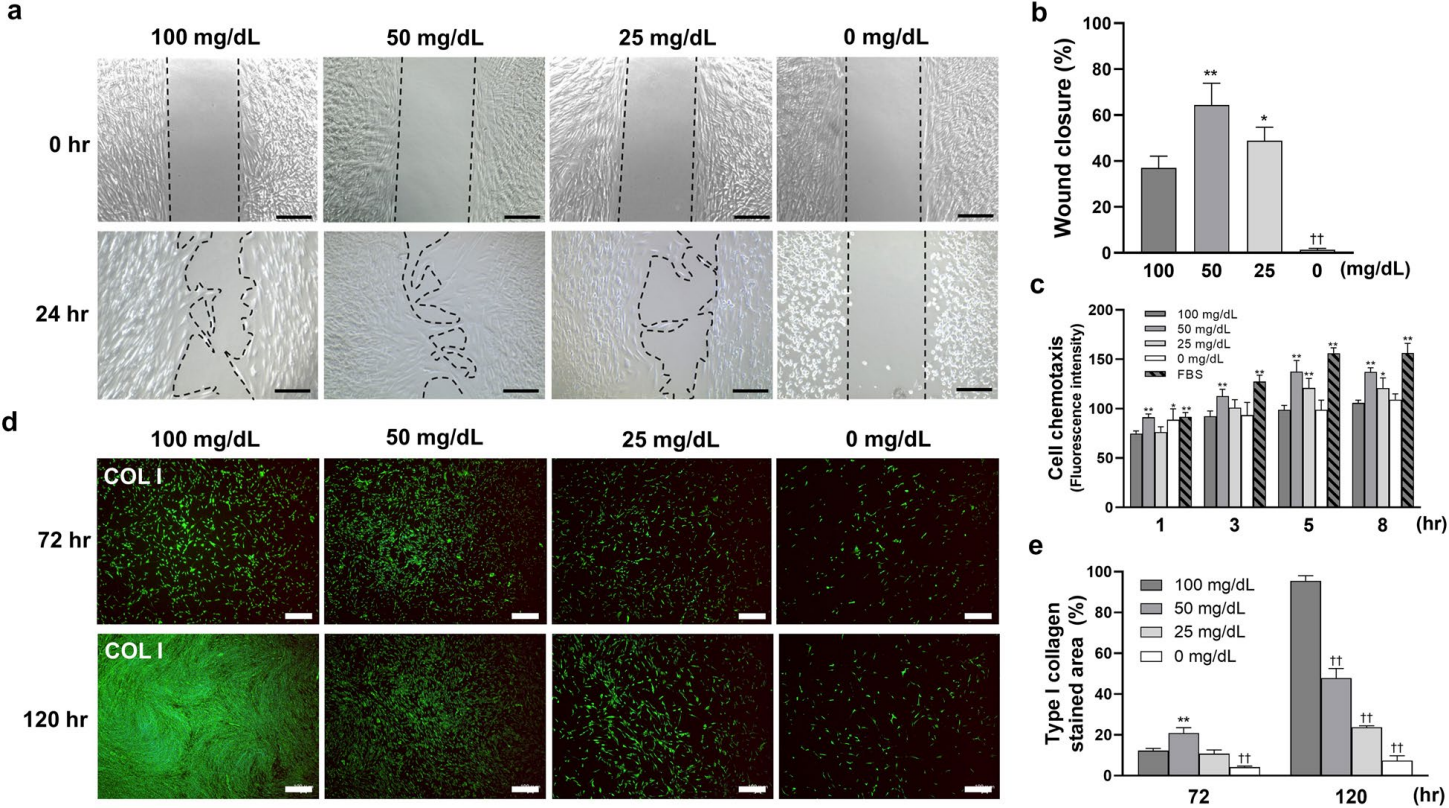
Low glucose concentrations affect the capability of wound-healing on human gingival fibroblast (HGnFs). (**a**) The process of wound healing assay was photographed at 0 and 24 h. **(b)** The data of wound healing assay is presented as the ratio of the final to the initial scratch size. **(c)** HGnFs cell migration under low glucose was indicated by fluorescence intensity. FBS (10%) was used as the positive control. (**d** and **e**) Type I collagen synthesis by HGnFs under low glucose concentrations was measured after incubation for 72 and 120 h. HGnFs were photographed after being incubated with fluorescently labeled secondary antibody and stained with DAPI. The data of the synthesis of type I collagen are presented as the ratio of the stained area. (Scale bars: 500 μm; A significant increase compared with the control was described as *P < 0.05, ** P < 0.01; A significant decrease compared with the control was described as ^††^*P* < 0.01).

Cell migration results are shown in Fig. 2a–c. Glucose concentrations of 50 and 25 mg/dL significantly promoted cell migration, with the highest migration observed in 50 mg/dL. By contrast, incubation with 0 mg/dL significantly inhibited cell migration.

The synthesis of type I collagen from HGnFs was showed in Fig. 2d,e after culturing for 72 and 120 h. The results showed that the synthesis was significantly increased in 50 mg/dL at 72 h. At 120 h, the synthesis was inhibited proportionally.

### Cellular stress level was increased in low glucose conditions as glucose decreased

**Figure 3:**
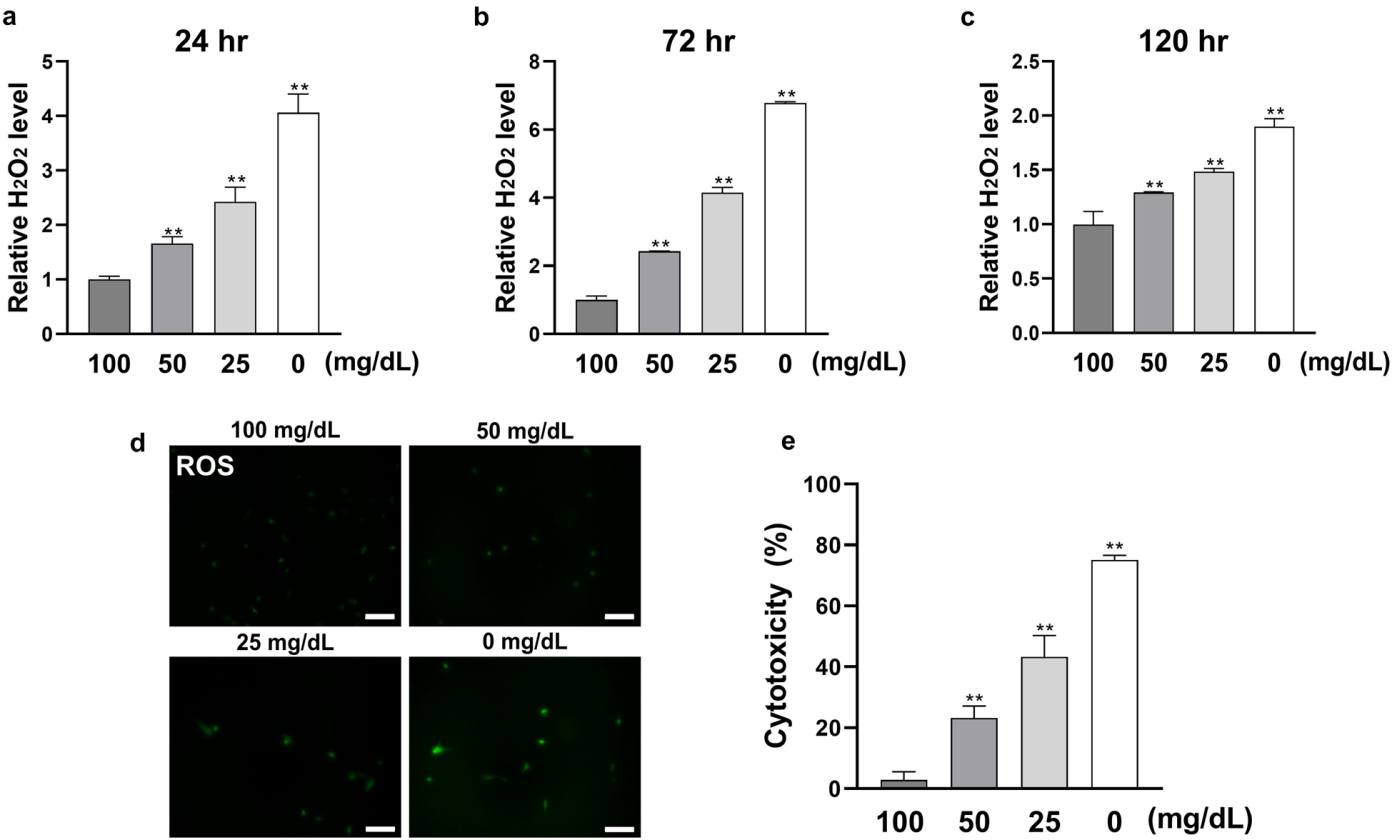
Low glucose concentrations enhance cellular stress level of HGnFs. (**a**, **b** and **c**) The level of ROS was examined using hydrogen peroxide (H_2_O_2_) detection kit. After 24, 72 and 120 h of incubation, the supernatant was collected and the level of H_2_O_2_ was measured by a microplate reader, and the results was modified relative to the control group. (**d**) The ROS stained cells were photographed by a fluorescence microscope after incubation for 120 h. (**e**) Cytotoxicity (LDH level) was determined after 24 h of incubation. (Scale bars: 100 μm; A significant increase compared with the control was described as **P < 0.01).

Hydrogen peroxide (H_2_O_2_) as a marker of ROS was measured after culturing for 24, 72, and 120 h. As shown in Fig. 3, the H_2_O_2_ level was significantly increased in all the stimulated groups (50, 25, and 0 mg/dL), with the peak H_2_O_2_ level recorded at 120 h. The intracellular ROS levels also increased proportionally in all the stimulated groups at 120 h. Hence, cytotoxicity increased proportionally in all stimulated groups.

### Glucose uptake capability was promoted in low glucose environment as glucose decreased

**Figure 4:**
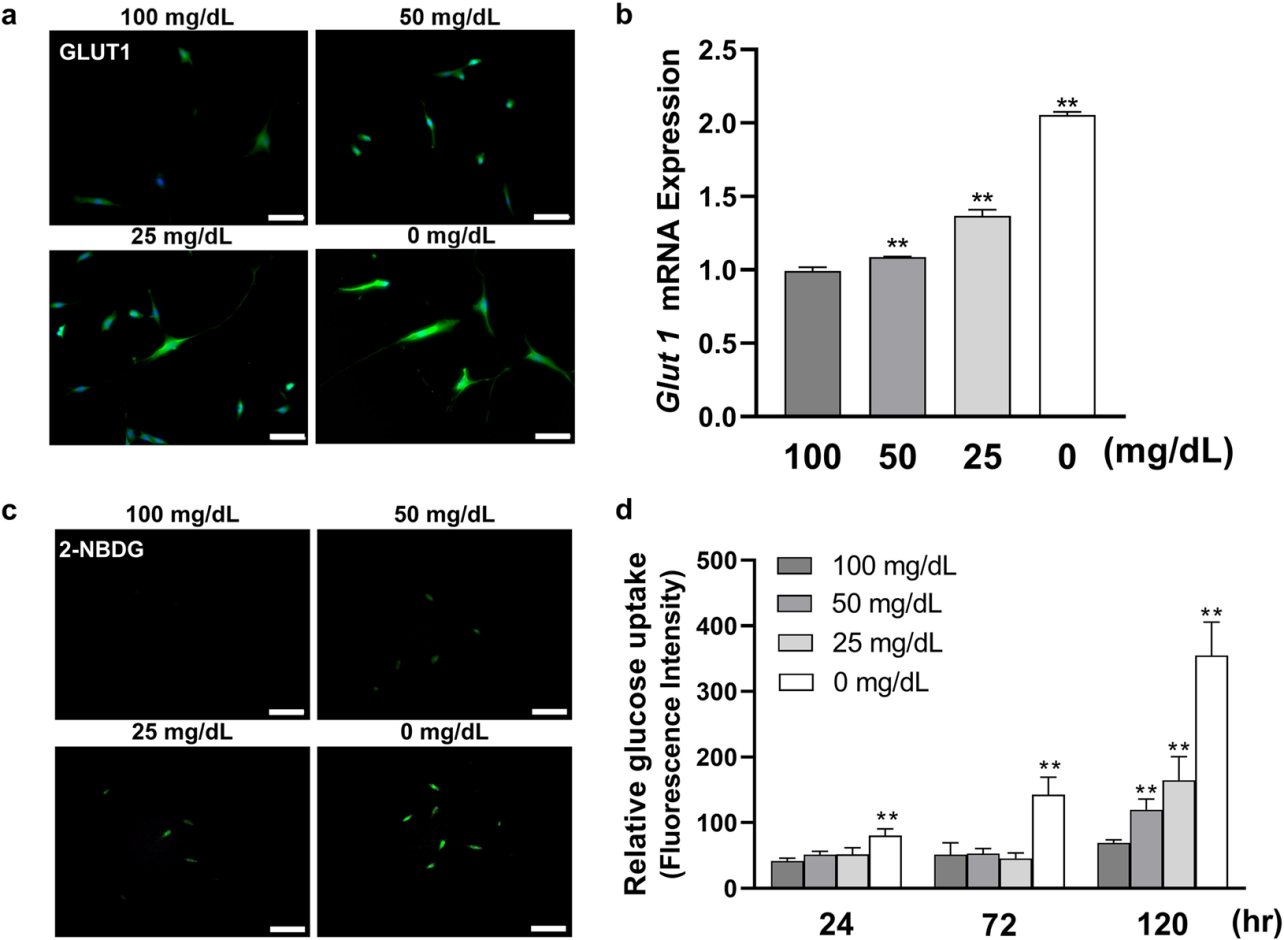
Low glucose concentrations improved glucose uptake capability on HGnFs. (**a** and **b**) The gene expression of GLUT1 was determined using PCR in stimulated HGnFs incubated for 72 h. HGnFs were photographed by a fluorescence microscope after being incubated with fluorescently labeled secondary antibody, and the nuclei were dyed with DAPI. (**c** and **d**) The capability of glucose uptake was measured by glucose uptake assay. After 24, 72 and 120 h of incubation, HGnFs were exposed to a medium with 100 μM 2-NBDG for 2 h, and the intensity of fluorescence was measured by a microplate reader and normalized by DNA. The stained cells were photographed by a fluorescence microscope. (Scale bars: 100 μm; A significant increase compared with the control was described as **P < 0.01).

The effects of low glucose on GLUT1 expression in HGnFs were shown in Fig. 4, analyzed by Real-time PCR and immunofluorescence staining. The expression of GLUT1 was significantly increased in the stimulated groups after 72 h. The uptake of glucose under low-glucose conditions on HGnFs was measured after culturing for 24, 72, and 120 h. As shown in Fig. 4c,d, the relative 2-NBDG uptake was enhanced proportionally in all stimulated groups.

### Glycolysis level was generally inhibited after once promoted

**Figure 5:**
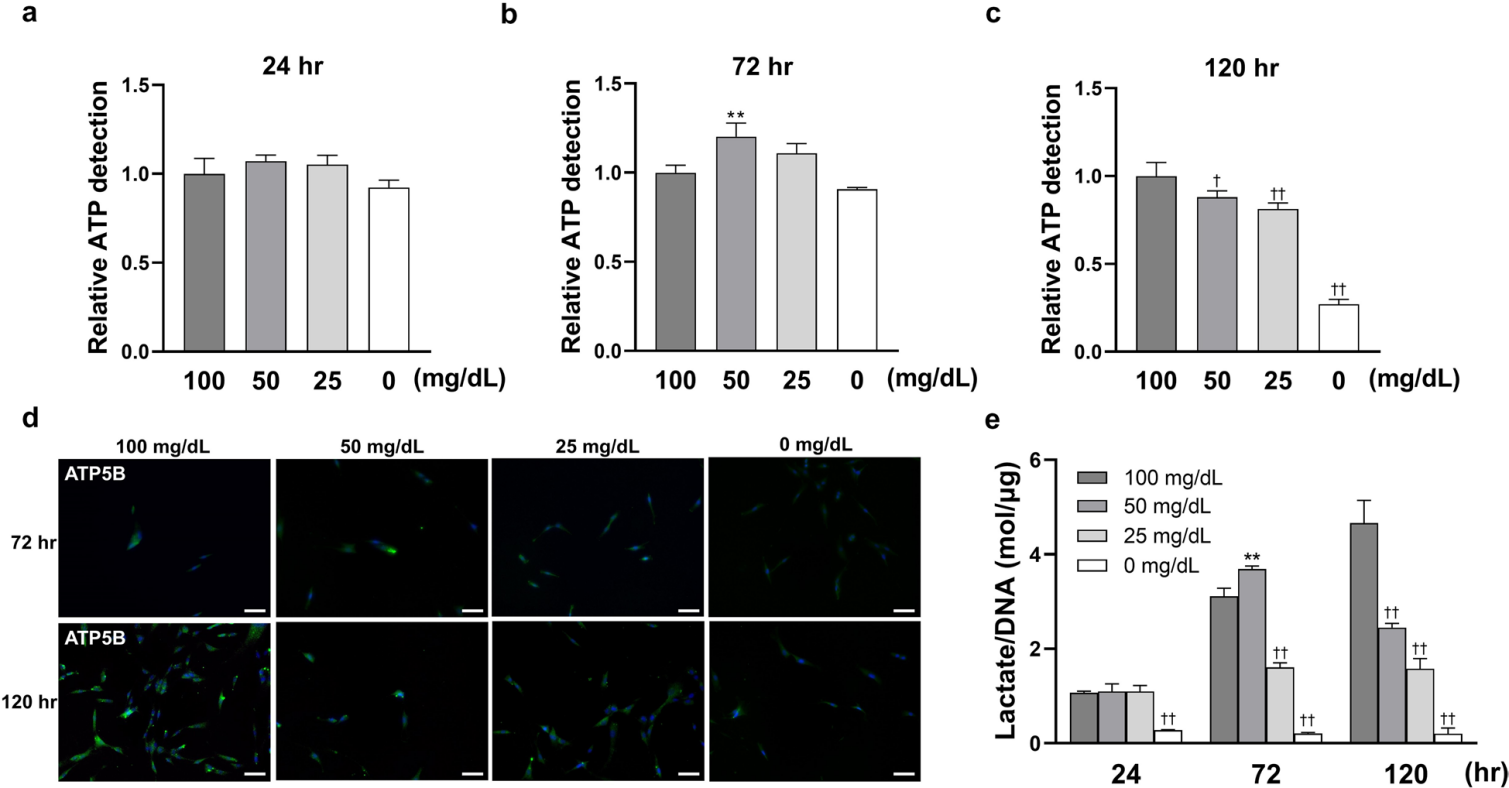
Low glucose concentrations affect glycolysis levels on HGnFs. (**a**, **b** and **c**) The level of intracellular ATP was determined by luminescence using a microplate reader after incubation for 24, 72 and 120 h, and the data was modified relative to the control group. (**d**) The activity of ATP5B was photographed by a fluorescence microscope. (**e**) Lactate production by HGnFs under low glucose was measured and normalized by DNA after incubation for 24, 72 and 120 h. (Scale bars: 100 μm; A significant increase compared with the control was described as **P < 0.01; A significant decrease compared with the control was described as ^†^*P* < 0.05, ^††^*P* < 0.01).

Lactate production and intracellular ATP levels in low-glucose condition were measured after culturing for 24, 72, and 120 h. As Fig. 5 showed that, lactate production was significantly increased in 50 mg/dL at 72 h; however, at 120 h, lactate production was proportionally decreased in all stimulated groups. By contrast, lactate production was significantly decreased at 0 mg/dL.

The intracellular ATP levels were significantly increased in 50 mg/dL at 72 h, and at 120 h, the intracellular ATP levels decreased in all stimulated groups. The activity of ATP5B was improved in 50 and 25 mg/dL at 72 h, with the highest increase detected in 50 mg/dL. After 120 h, ATP5B activity in stimulated groups were significantly reduced compared with 100 mg/dL group.

### Low glucose activated AMPK and induced autophagy

**Figure 6:**
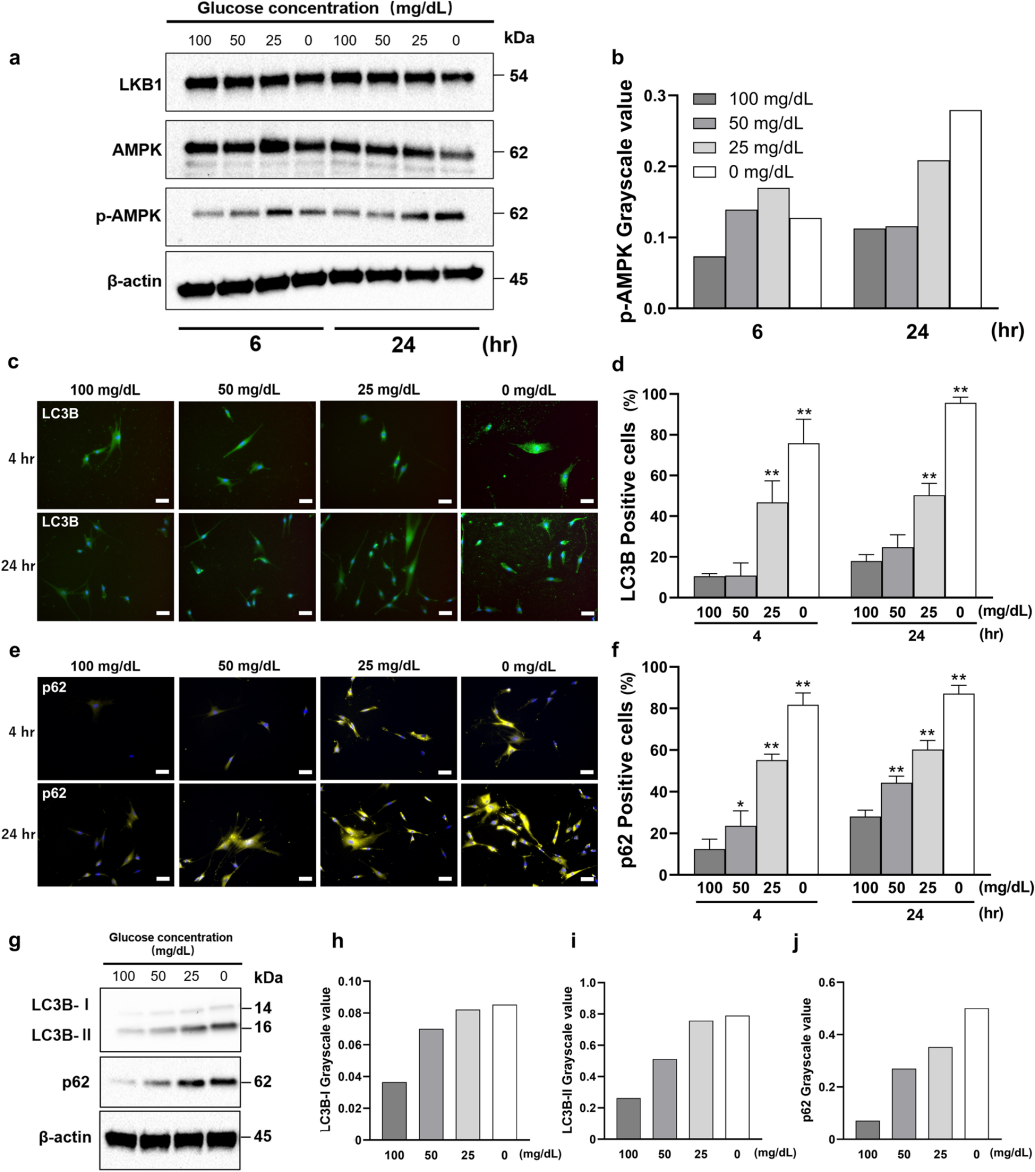
Low glucose concentrations activate the LKB1-AMPK signaling pathway and induce autophagy. (**a** and **b**) The protein expression of LKB1-AMPK signaling pathway was evaluated (**a**) using western blotting analysis and quantified using ImageJ (**b**). Western blot analysis was performed on protein extracts of these cells with antibodies against the indicated proteins with β-actin as a loading control. The samples derive from the same experiment and those gels/blots were processed in parallel. Uncropped blots for this experiment are presented in supplementary file. The expression levels of LKB1, AMPK and p-AMPK were measured by densitometric analysis using ImageJ. (**c**–**j**) The synthesis of autophagy-related proteins (LC3B and p62) by HGnFs under low glucose concentrations. HGnFs were photographed after being incubated with fluorescently labeled secondary antibody and stained with DAPI (**c** and **e**). The data of the synthesis of LC3B and p62 are presented (**d** and **f**) as the ratio of the positive cells. The expression of LC3B and p62 were examined using western blotting (**g**) and analyzed using ImageJ (**h**, **i**, and **j**). Western blot analysis was performed on protein extracts of these cells with antibodies against the indicated proteins with β-actin as a loading control. The samples derive from the same experiment and those gels/blots were processed in parallel. Uncropped blots for this experiment are presented in supplementary file. The expression levels of p62, LC3B-I and LC3B-II were measured by densitometric analysis using ImageJ. (Scale bars: 100 μm; A significant increase compared with the control was described as *P < 0.05, **P < 0.01).

We showed the change of HGnFs function, such as the autophagy by using western blotting and immunofluorescence staining. As shown in the Fig. 6, low glucose increased the protein expression levels of p-AMPK, with the greatest changes observed at 0 mg/dL. After stimulation for 4 h and 24 h, the expression of autophagy-related proteins (LC3B and p62) was significantly increased.

## Discussion

This in-vitro study investigated the effects of low glucose environments on gingival wound healing using HGnFs. Our results showed that different glucose concentrations induced different cellular functions, such as cell proliferation, migration, and synthesis of type I collagen. Furthermore, our novel finding is that extremely low glucose environments induce cellular stress and autophagy in HGnFs.

Glucose starvation can inhibit cell proliferation^13^. This is comparable to our finding that the proliferation and migration of HGnFs were inhibited in the absence of glucose (0 mg/dL group). Moreover, low glucose (20 mg/dL) could promote the migration of cancer cells^14^. We observed a similar phenomenon in all migration assay. Furthermore, low glucose concentration may inhibit the synthesis of collagen in fibroblasts^15^; this was also consistent with our findings. Our original hypothesis was that as the glucose concentration decreases, cell proliferation and migration would gradually decrease as well. However, during the first 72 h, cell proliferation in the 50 and 25 mg/dL groups increased compared to that in the control (100 mg/dL) group. At 120 h, the proliferation decreased in all stimulated (50 ,25, 0 mg/dL) groups. Moreover, cell migration and type I collagen synthesis showed the same trend as that of proliferation. These results indicated that the gingival wound healing capability was inhibited by low glucose after 120 h but improved in 50 mg/dL glucose concentration after 72 h. The possible reason for this phenomenon is that the wound healing capacity of HGnFs is activated when the glucose concentration in the environment is lower than the physiological glucose concentration, but this activation mechanism is then inhibited by the extremely low glucose environment. As the cells proliferate (the number of cells becomes greater), the rate of glucose consumption in the environment increases, and when the glucose concentration falls under a certain threshold (extremely low glucose concentration), the activation of the cells is inhibited.

We first evaluated the cellular stress factor (ROS and LDH) levels of HGnFs under low-glucose conditions. Cellular stress factors, such as ROS and LDH, could inhibit fibroblast proliferation and migration and delay wound healing in the connective tissue^16^. In addition, the release of LDH into the culture medium is a marker of cytotoxicity^17^. Our results showed that ROS and cytotoxicity levels were significantly increased under low-glucose conditions. Furthermore, we found that the cellular stress level was positively correlated with a decrease in glucose. This suggests that the lower the level of glucose, the greater the cellular stress on HGnFs. Interestingly, even when the cytotoxicity level was as high as 75%, cell death was not confirmed. A study has presented the same trend, which is comparable to our results^18^. Based on these results, we found that HGnFs can survive without glucose for some time during stress responses. The oral environment is one of the severest environments of the human body. There are more than 700 bacterial species living in the oral environment and the temperature in the mouth can change dramatically depending on the temperature of the food being eaten^19,20^. Due to this, the capability of the cells to maintain homeostasis in oral tissue is particularly important. In this study, we explored the effects of cellular stress caused by low glucose concentrations on HGnFs. We found that HGnFs can survive for a period of time under low glucose pressure, which is important for maintaining the homeostasis of the oral environment.

Glucose metabolism consists of two processes: glucose uptake and glycolysis. In the process of glucose uptake, transportation across the cellular membrane is the main rate-limiting step, which is determined by facilitated-diffusion GLUTs^21^. Of all the GLUTs, GLUT1 is responsible for the low level of basal glucose uptake required to maintain respiration in all cells ^22^. In our study, low glucose enhanced the expression of GLUT1 in HGnFs, which suggests that low glucose could enhance glucose uptake proportionally as HGnFs sense glucose deprivation. 2-NBDG is a fluorescent tracer used to monitor glucose uptake in living cells ^23^. We observed that low glucose could improve the uptake of 2-NBDG, indicating that the glucose uptake potential of HGnFs was proportionally enhanced by low glucose conditions. These results indicate that when a low-glucose environment is sensed, HGnFs enhances its glucose uptake by increasing GLUT1 expression, thereby indicating a negative correlation between glucose concentration and its uptake. Glycolysis is a complex biochemical process mediated by multiple glycolytic genes and pathways. Lactate is an intermediate cellular metabolite of the glycolytic pathway, and is produced in the cytoplasm through the reduction of the pyruvate ^24^; Meanwhile, ATP is the end metabolite of glycolysis^25^. Thus, their levels may reflect the level of glycolytic activity of the cell^26^. Our result showed that lactate and ATP production was relatively increased in the 50 mg/dL group at 72 h, which could be an indication of enhanced glycolytic activity. However, a decrease was observed in lactate and ATP production compared to that in the control group at 120 h. This result indicated that the glucose metabolism in the 50 mg/dL group was higher than that in the control group in the first 72 h, which could explain the enhanced wound healing capacity of 50 mg/dL group at 72 h. And the result of 120 h showed that glucose metabolism was reduced in all stimulated groups, which may indicate that glucose uptake was not sufficient to support consumption levels compared to the control group, ultimately leading to a suppression of wound healing. By contrast, we found that a small amount of ATP was produced in the group without glucose.

To further explain why cell death was not detected and ATP was produced even under glucose deprivation, we investigated the HGnF autophagy under low-glucose conditions. Previous studies have shown that ROS can induce autophagy in cells through the LKB1-AMPK signaling pathway to prevent damage without inducing cell death^27^. Autophagy is a regulatory mechanism of the cell that removes dysfunctional or unnecessary cellular components or proteins, thereby allowing for their systematic degradation and recycling^28^. It is often seen as an adaptive response to stress, used to maintain cellular energy levels and to promote cell survival. It is reported that autophagy can be induced by bacteria or inflammation in periodontal tissues^29,30^. LC3B and p62 (sequestosome 1 [SQSTM1]) have been investigated as markers of autophagy^28^. We observed that low glucose concentrations increased the expressions of LC3B and p62. This result may indicate that autophagy of HGnFs can be induced by extremely low glucose levels alone without the presence of inflammation or bacteria, thus maintaining cell survival and preventing cell death. This provides a possible explanation for no detectable cell death (from PI staining) throughout the experiment.

There is a close relationship between cell death and autophagy. As some studies have shown, the same gene controls both apoptotic cell death and autophagy. The autophagic signaling pathway can regulate apoptotic cell death, while apoptosis could also regulate autophagy^31^. In this study, we found that autophagy caused by low glucose condition may inhibited cell death. Thus, we assume that the autophagy plays a key role in glucose starvation both for pro-survival and prevent the cell death. However, the molecular connection between autophagy and cell death remains unclear. In future study, we will explore more about the relationship between autophagy and cell death of HGnFs in glucose starvation condition.

Based on a series of results, including the capacity of wound healing, cellular stress, glucose metabolism, and autophagy, we hypothesized that the LKB1-AMPK signaling pathway plays an important role in these processes. This pathway senses intracellular energy levels and ensures energy supply and cell survival by saving or producing ATP when energy is insufficient^11^. Thus, it may be essential for cell survival under glucose starvation. Moreover, this pathway can be activated not only by glucose starvation but also by ROS^32^. AMPK can also promote glucose utilization by phosphorylating targets involved in GLUT1 and GLUT4 transport^12^. In addition, the phosphorylation of AMPK could also increase the phosphorylation of thioredoxin-interacting protein (TXNIP13), which could enhance the expression of GLUT1, thus improving glucose uptake^33^. Moreover, AMPK activation triggers autophagy. We observed that low glucose levels proportionally activated AMPK, which may explain the resulting elevated glucose metabolism capacity and induced autophagy.

Clinically, the repair of wounds in the gingival tissue is faster compared to that in other body tissues. Even when large gingival incisions are performed during periodontal surgery, they heal quickly within a few days. We observed that even in an extremely low-glucose environment, the cells survived by inducing autophagy to produce ATP by themselves. This novel finding may contribute to a deeper understanding of the mechanisms of gingival soft tissue healing and may explain the reason behind the rapid healing of gingival tissue after periodontal surgery.

Although the cell biology and mechanisms of gingival wound healing have been well studied, there is much uncertainty regarding the relationship between the wound environment nutrition and healing. We designed and investigated an in vitro gingival wound healing model that simulates a low-nutrient microenvironment through glucose deprivation, because glucose is one of the most important ingredients of all nutrients^34^. However, the limitation of this concept is that several other factors in the wound microenvironment, such as serum and blood oxygen levels, were not simulated due to its in-vitro design. And in animal experiments, if the blood glucose level is artificially lowered, the animals themselves will die due to hypoglycemia and the experimental procedure will not be reproducible. Therefore, further studies are needed and these variables can be considered.

In this study, we found that low glucose (50 mg/dL) enhances wound healing by improving cellular glucose uptake through the LKB1-AMPK signaling pathway. By contrast, an extremely low glucose level (25 or 0 mg/dL) inhibits wound healing and induces ROS and autophagy. These findings may contribute to a better understanding of gingival wound healing during glucose starvation, such as immediately after periodontal surgery.

## Materials and methods

### Cell culture

HGnFs were obtained from ScienCell Research Laboratories (San Diego, CA, USA). HGnFs were incubated in Dulbecco's modified eagle medium (DMEM) (Nacalai Tesque, Kyoto, Japan) with four glucose concentrations: 100 (normal control), 50, 25, and 0 mg/dL, and these were prepared by mixing two types of DMEM (100 mg/dL and 0 mg/dL).

### Cell proliferation assay

Proliferation data were determined using Cell Count Reagent SF (Nacalai Tesque). The data were analyzed using SoftMax® Pro Microplate Data Acquisition and Analysis software (Version 7.0, Molecular Devices, Sunnyvale, CA, USA).

For live and dead staining, HGnFs were stained with calcein-acetoxymethylester (calcein-AM) and propidium iodide (PI) solutions, respectively (Dojindo, Kumamoto, Japan). The stained cells were photographed using a BZ-II all-in-one fluorescence microscope (Keyence Corporation, Osaka, Japan).

### Cell migration assay

The modified Boyden chamber assay was performed using micro chemotaxis chambers (Fluoroblock™ insert system, Falcon, Corning, NY, USA), following the manufacturer’s instructions. Wound-healing assays were performed using a wound-repair assay kit (Ibidi GmbH, Am Klopferspitz 19, Martinsried, Germany), following the manufacturer’s instructions.

### Immunofluorescence staining

Following stimulation, the cells were incubated with primary antibodies against human type I collagen (Abcam, Cambridge, UK), glucose transporter type 1 (GLUT1) (Abcam), ATP5B (Santa Cruz Biotechnology, Inc. Dallas, Texas, USA), LC3B and p62 (MBL, Tokyo, Japan). Fluorescent immunostaining was performed using Alexa Fluor 488® (Thermo Fisher Scientific, Inc., Waltham, MA, USA) as the secondary antibody, and the nuclei were stained using 4',6-diamidino-2-phenylindole (DAPI) (Dojindo). Images of stained cells were obtained using the fluorescence microscope. These images were then analyzed.

### ROS detection and cytotoxicity assay

ROS levels were measured using a hydrogen peroxide fluorometric detection kit and ROS-ID® Hypoxia/Oxidative Stress Detection Kit (Enzo Life Sciences, Farmingdale, NY, USA), and lactate dehydrogenase (LDH) activity was measured using the Cell Cytotoxicity LDH Assay Kit-WST (Dojindo), following the manufacturer’s instructions.

### Gene expression in HGnFs

Total RNA was isolated using the RNeasy Mini Kit (Qiagen, Venlo, Netherlands). RNA from each sample was reverse-transcribed to cDNA using the PrimeScript Reagent Kit (Takara Bio Inc, Shiga, Japan). The gene expression of GLUT1 (TaqMan® Gene Expression Assay; Applied Biosystems™, Thermo Fisher Scientific) was quantified using the StepOnePlus™ Real-Time PCR System (Thermo Fisher Scientific).

### Determination of glucose uptake, lactic acid production, and ATP level

After stimulation, 2-[N-(7-nitrobenz-2-oxa-1, 3-diazol-4-yl) amino]-2-deoxy-D-glucose (2-NBDG) (Invitrogen™, Thermo Fisher Scientific) was added to the medium. Fluorescence was photographed using fluorescence microscope, measured by a microplate reader, and then normalized to the quantity of DNA.

Lactaic acid production was measured using a Lactate Assay Kit-WST (Dojindo), according to the manufacturer’s instructions. HGnF were stimulated, the supernatants were collected for analysis, and the data were normalized to DNA. ATP levels were determined by luminescence using the CellTiter-Glo 2.0 luminescent cell viability assay (Promega, Madison, Wisconsin, USA), following the manufacturer’s instructions.

### Western blot analysis

Proteins were extracted using RIPA buffer (Thermo Fisher Scientific) supplemented with a protease inhibitor cocktail (Thermo Fisher Scientific) and phosphatase inhibitor cocktail (Nacalai Tesque) and normalized using BCA Protein Assay kit (Thermo Fisher Scientific). Protein samples were separated by 12.5% SDS-PAGE (Nacalai Tesque) and transferred onto polyvinylidene difluoride membranes (Bio-Rad, Hercules, CA, USA). Then, the membranes were blocked using Blocking One (Nacalai Tesque) and incubated with primary antibodies against AMPKα, p-AMPKα (Thr172), LKB1, light chain 3B (LC3B), β-actin (Cell Signaling Technology, Danvers, Massachusetts, USA), and p62 (MBL). The membranes were then washed and incubated with secondary antibodies. Immunoreactive bands were visualized using a chemiluminescence kit (Nacalai Tesque), and the signals and western blotting data were analyzed using ImageJ Ver. 1.53e (Wayne Rasband and contributors, National Institutes of Health, USA, http://rsb.info.nih.gov/ij).

### Statistical analysis

Data were analyzed and are presented as mean ± SD. Parametric data were analyzed using one-way analysis of variance (ANOVA) with Tukey's test by IBM SPSS Statistics Ver. 17 (IBM, Chicago, IL, USA).

## Supplementary Information

Supplementary Information.

## Data Availability

The datasets generated during and/or analysed during the current study are available from the corresponding author on reasonable request.
