# Supplementary material for: Intracellular glucose starvation affects gingival homeostasis and autophagy
Source: Sci Rep. 2022 Jan 24;12:1230. doi: 10.1038/s41598-022-05398-2 (PMC8786886; doi:10.1038/s41598-022-05398-2)
Supplement: Supplementary file 1 — Supplementary Information. [file 41598_2022_5398_MOESM1_ESM.pdf]

Supplementary file
Title page

Article title Intracellular glucose starvation affects gingival homeostasis and autophagy

Supplementary item: 20 Figures

Author information
Runbo Li; Department of Periodontology, Osaka Dental University, Osaka Japan
E-mail: li-r@cc.osaka-dent.ac.jp
Hirohito Kato; Department of Periodontology, Osaka Dental University, Osaka Japan
E-mail: kato-h@cc.osaka-dent.ac.jp
Yoichiro Taguchi; Department of Periodontology, Osaka Dental University, Osaka Japan
E-mail: taguchi@cc.osaka-dent.ac.jp
Makoto Umeda; Department of Periodontology, Osaka Dental University, Osaka Japan
E-mail: umeda-m@cc.osaka-dent.ac.jp

Corresponding Author: Yoichiro Taguchi
Department of Periodontology, Osaka Dental University
8-1, Kuzuhahanazono-cho, Hirakata, Osaka, Japan
TEL: +81-72-864-3084
FAX: +81-72-864-3184
E-mail: taguchi@cc.osaka-dent.ac.jp

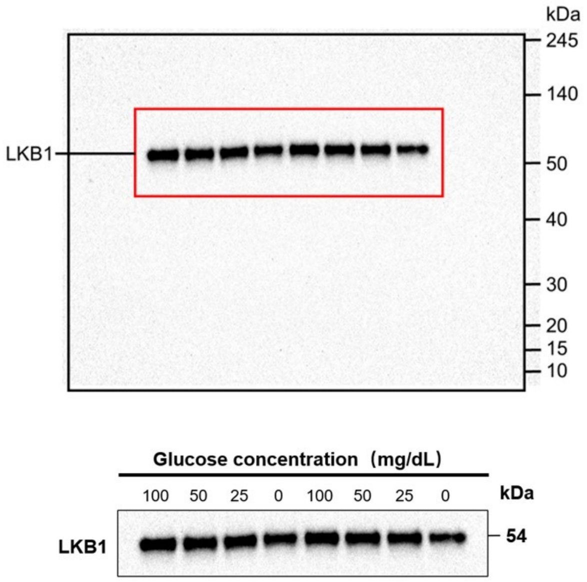

Figure S1: The original whole western blot of LKB1 (Figure 6a, on the panel below) exposed for 300s. For the final figure, the regions of the original blots have been denoted using red boxes. PowerPoint was used to crop the excess outside the red boxes.

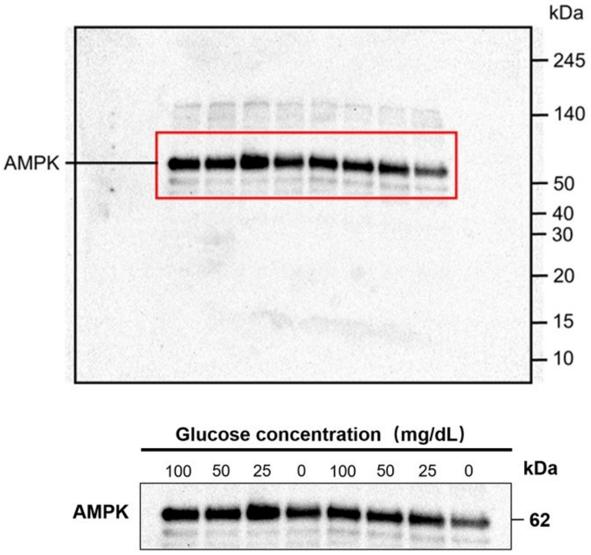

Figure S2: The original whole western blot of AMPK (Figure 6a, on the panel below) exposed for 300s. For the final figure, the regions of the original blots have been denoted using red boxes. PowerPoint was used to crop the excess outside the red boxes.

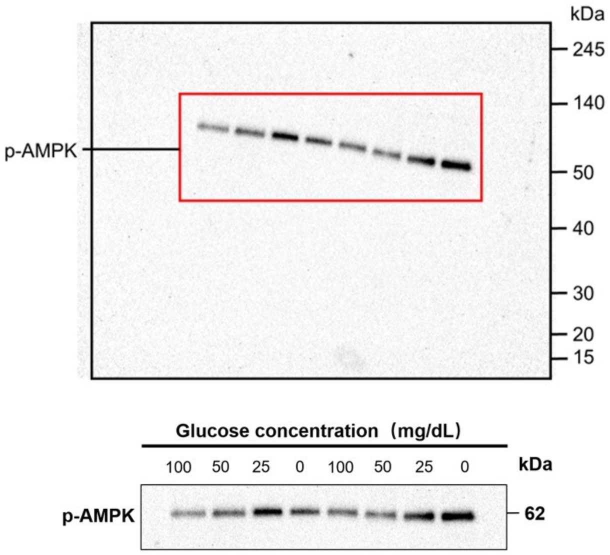

Figure S3: The original whole western blot of p-AMPK (Figure 6a, on the panel below) exposed for 320s. For the final figure, the regions of the original blots have been denoted using red boxes. PowerPoint was used to straight the blot and crop the excess outside the red boxes.

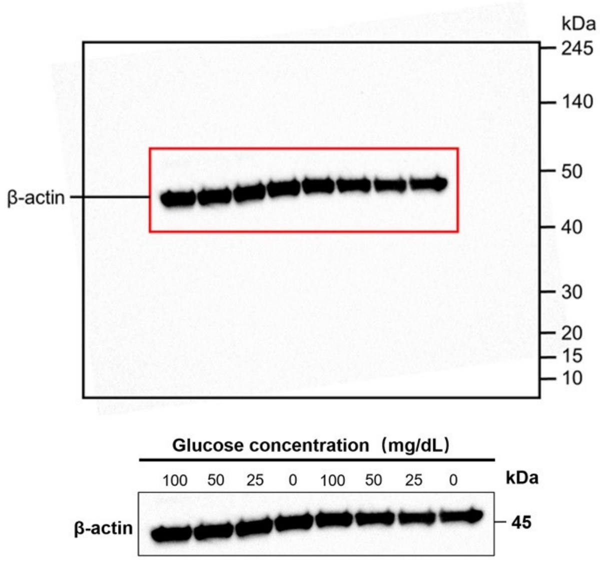

Figure S4: The original whole western blot of β-actin (Figure 6a, on the panel below) exposed for 30s. For the final figure, the regions of the original blots have been denoted using red boxes. PowerPoint was used to crop the excess outside the red boxes.

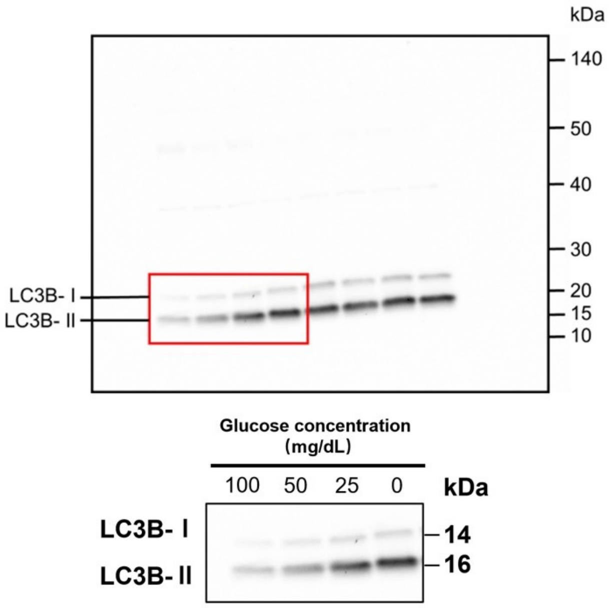

Figure S5: The original whole western blot of LC3B (Figure 6g, on the panel below) exposed for 150s. For the final figure, the regions of the original blots have been denoted using red boxes. PowerPoint was used to crop the excess outside the red boxes.

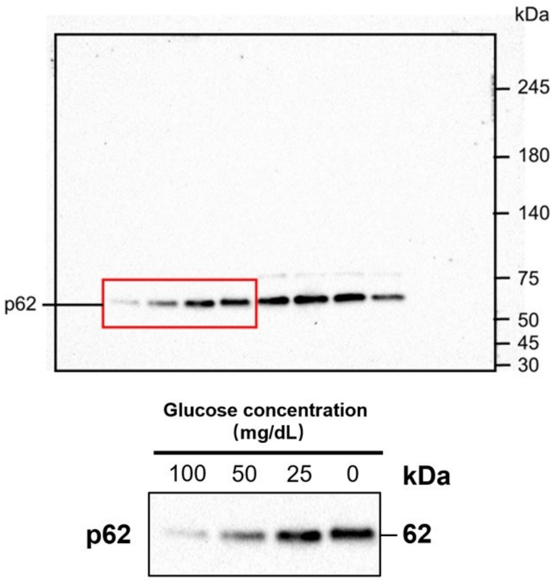

Figure S6: The original whole western blot of p62 (Figure 6g, on the panel below) exposed for 300s. For the final figure, the regions of the original blots have been denoted using red boxes. PowerPoint was used to crop the excess outside the red boxes.

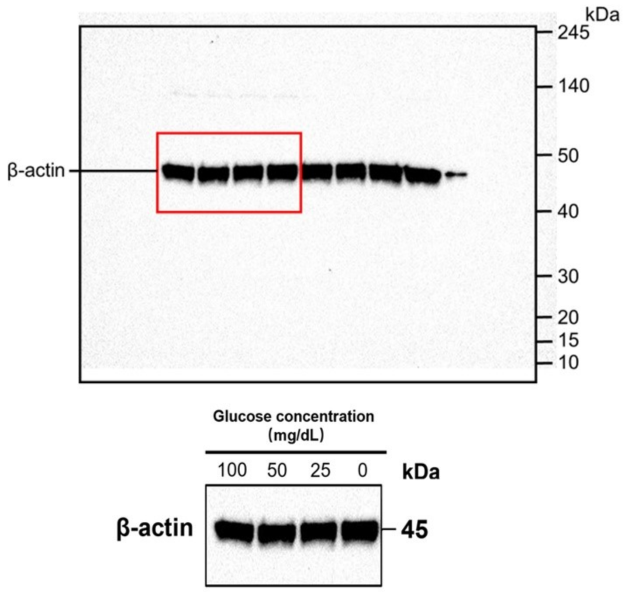

Figure S7: The original whole western blot ofβ-actin (Figure 6g, on the panel below) exposed for 30s. For the final figure, the regions of the original blots have been denoted using red boxes. PowerPoint was used to crop the excess outside the red boxes.

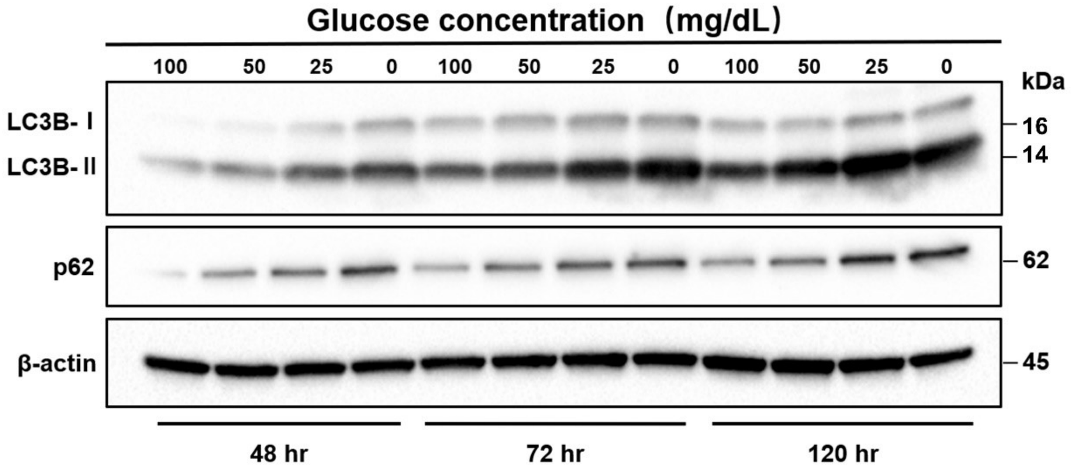

Figure S8: The western blots of long-time pattern autophagy obtained during the additional experiment conducted for reviewer 2.

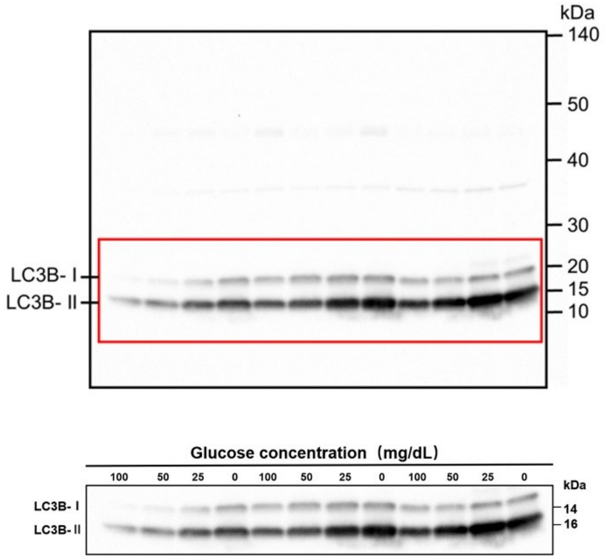

Figure S9: The original whole western blot of LC3B (Figure S8, on the panel below) obtained during the additional experiment conducted for reviewer 2 and exposed for 180s. For the final figure, the regions of the original blots have been denoted using red boxes. PowerPoint was used to crop the excess outside the red boxes.

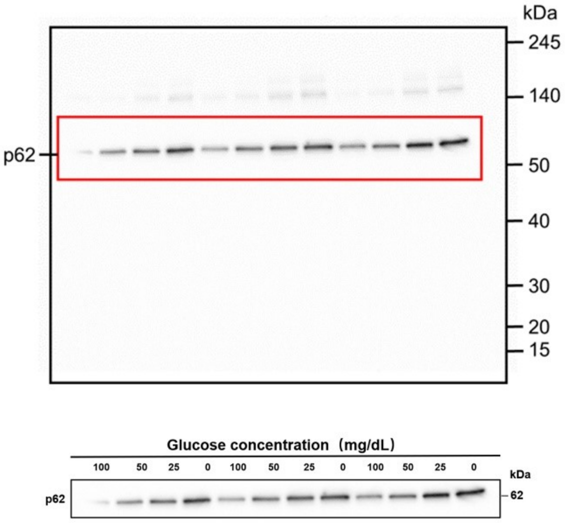

Figure S10: The original whole western blot of p62 (Figure S8, on the panel below) obtained during the additional experiment conducted for reviewer 2 and exposed for 60s. For the final figure, the regions of the original blots have been denoted using red boxes. PowerPoint was used

to crop the excess outside the red boxes.

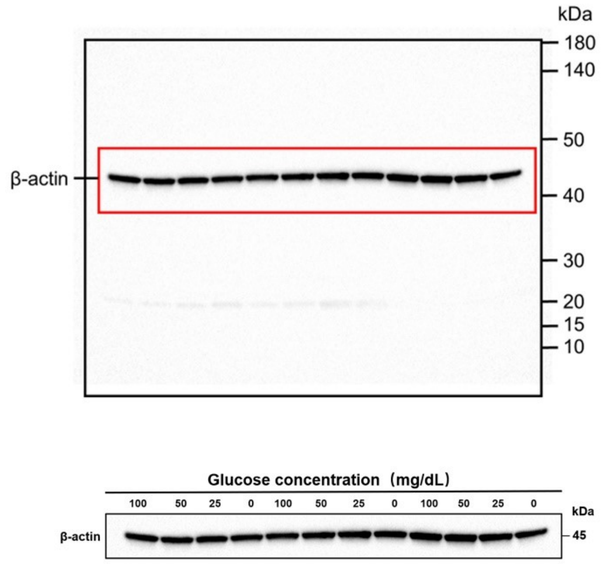

Figure S11: The original whole western blot of b-actin (Figure S8, on the panel below) obtained during the additional experiment conducted for reviewer 2 and exposed for 30s. For the final figure, the regions of the original blots have been denoted using red boxes. PowerPoint was used to crop the excess outside the red boxes.

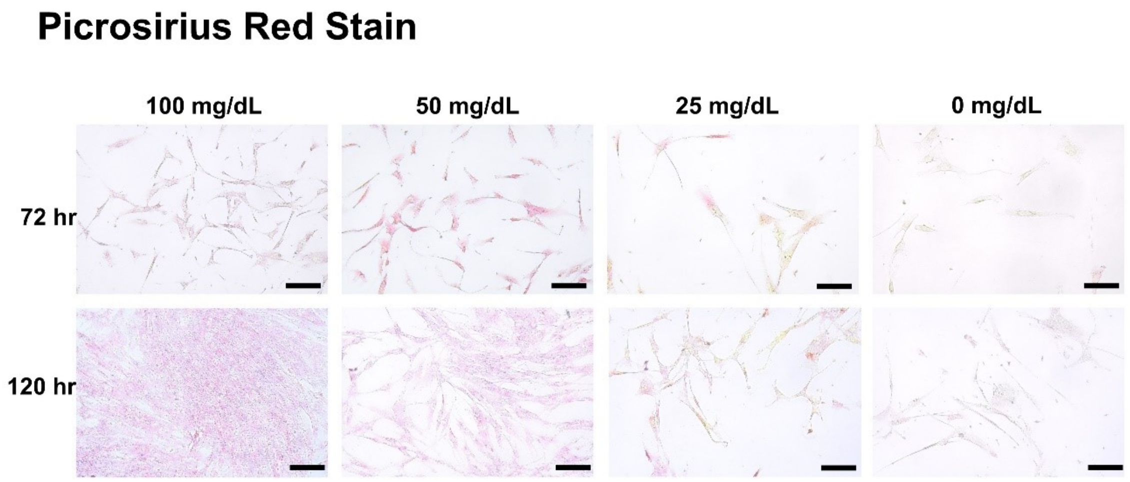

Figure S12: The Picrosirius red staining of type 1 collagen in HGnFs of low glucose conditions obtained during the additional experiment conducted for reviewer 1. (Scale bar :100 μm)

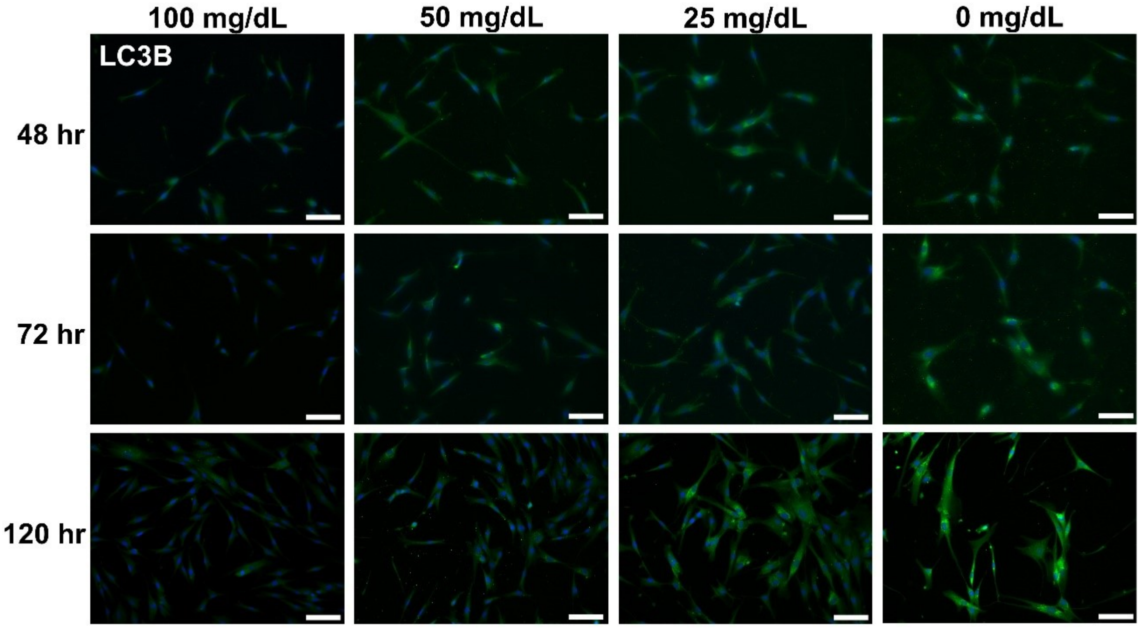

Figure S13: The Immunofluorescence staining of long-time pattern autophagy-related protein LC3B obtained during the additional experiment conducted for reviewer 2. (Scale bar :100 μm)

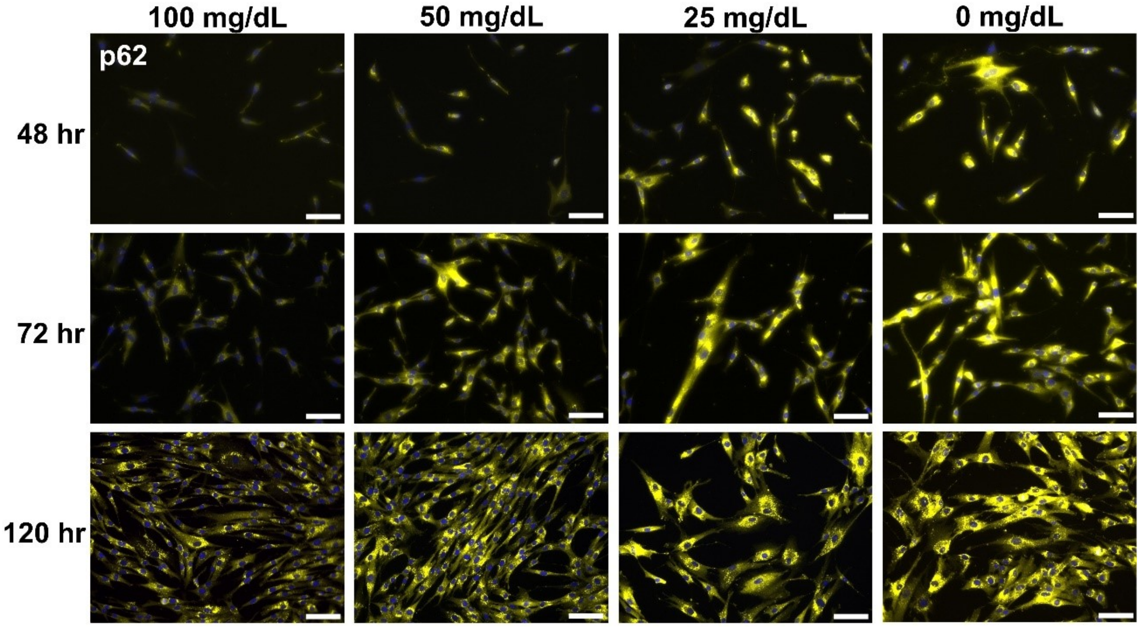

Figure S14: The Immunofluorescence staining of long-time pattern autophagy-related protein p62 obtained during the additional experiment conducted for reviewer 2. (Scale bar :100 μm)

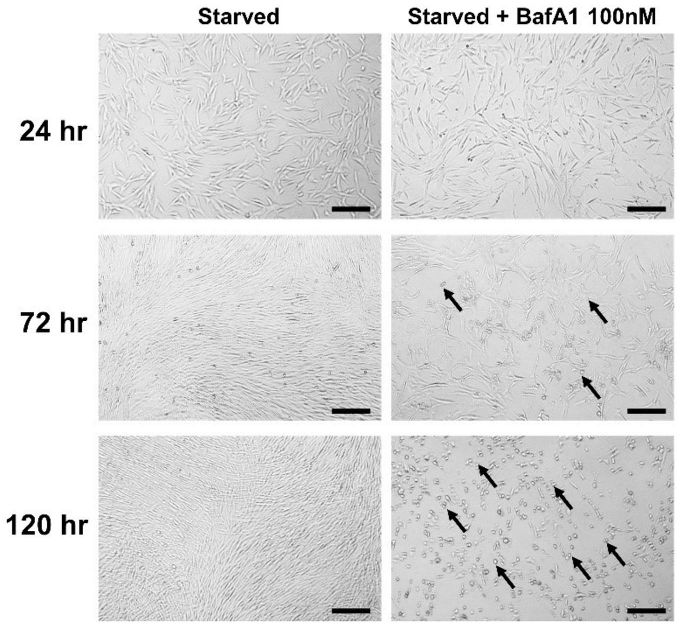

Figure S15: The light micrograph of starved cell incubates with and without bafilomycin A1 obtained during the additional experiment conducted for reviewer 2 (Scale bar: 50μm).

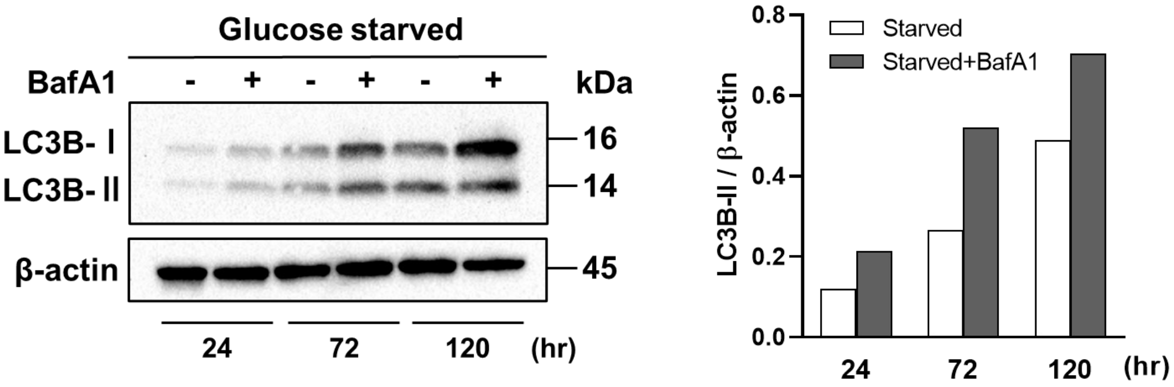

Figure S16: The western blots and the analysis of LC3 flux assay obtained during the additional experiment conducted for reviewer 2.

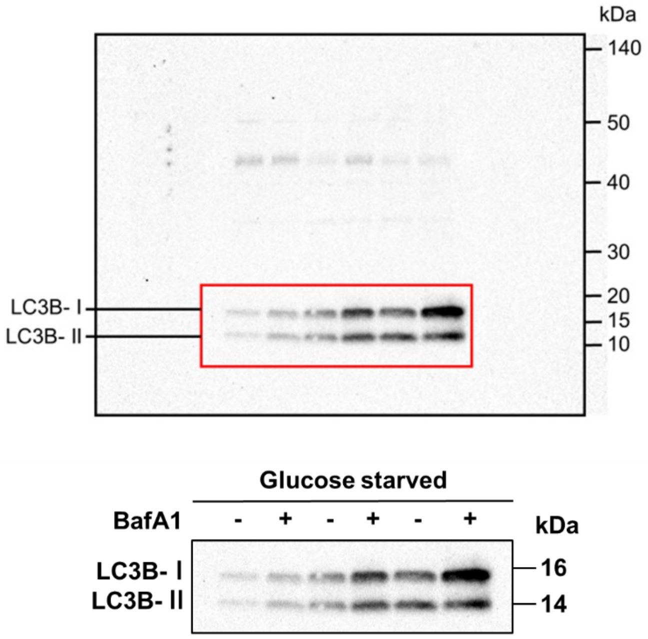

Figure S17: The original whole western blot of LC3B (Figure S15, on the panel below) obtained during the additional experiment conducted for reviewer 2 and exposed for 180s. For the final figure, the regions of the original blots have been denoted using red boxes. PowerPoint was used to crop the excess outside the red boxes.

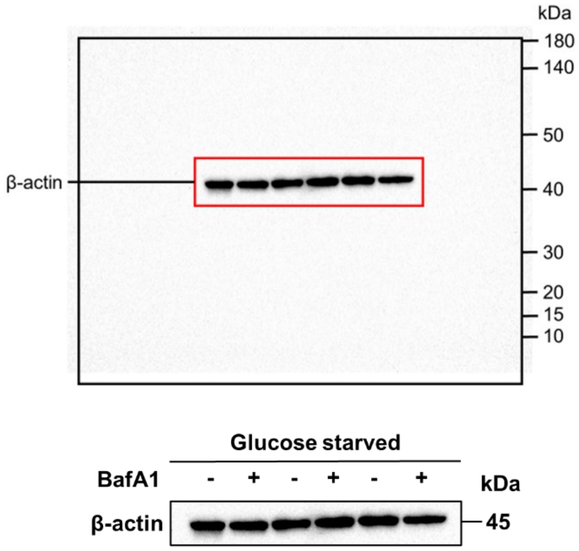

Figure S18: The original whole western blot of b-actin (Figure S15, on the panel below) obtained during the additional experiment conducted for reviewer 2 and exposed for 180s. For the final figure, the regions of the original blots have been denoted using red boxes. PowerPoint was used to crop the excess outside the red boxes.

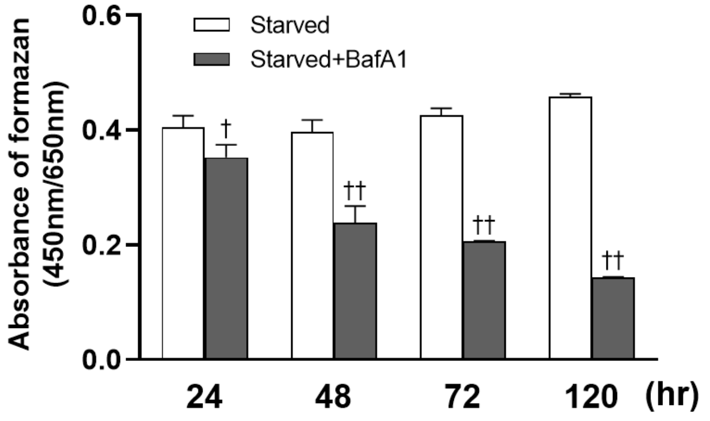

Figure S19: Cell proliferation in starved cell incubates with and without bafilomycin A1 obtained during the additional experiment conducted for reviewer 2.

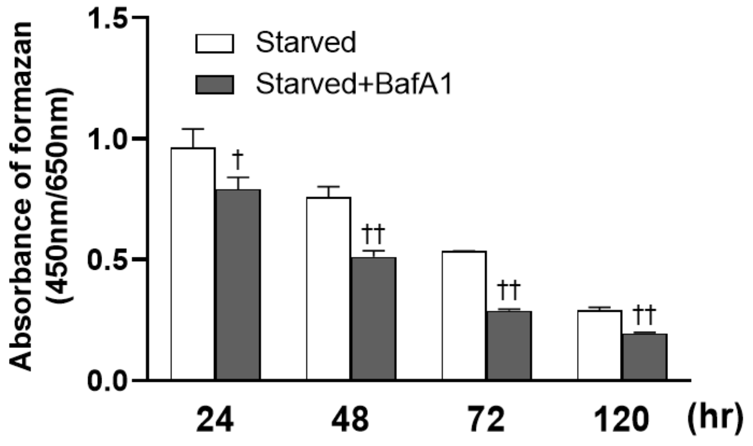

Figure S20: Cell proliferation in starved cell incubates with and without bafilomycin A1 obtained during the additional experiment conducted for reviewer 2.
